# Clinical leadership education—A scoping review protocol

**DOI:** 10.1111/aas.14591

**Published:** 2025-02-23

**Authors:** Naja Vaaben, Peter Dieckmann, Ann Merete Møller

**Affiliations:** ^1^ University of Copenhagen Copenhagen Denmark; ^2^ Herlev Hospital Herlev Denmark

**Keywords:** education, leadership, training

## Abstract

**Introduction:**

Leadership is a key skill for physicians, but studies show that many junior doctors feel unprepared for the role as a leader. In recent years, there has been increasing interest and research into the training and development of clinical leadership, revolving around leadership in situations with direct patient care and as opposed to the administrative leadership of a department. However, the lack of consensus on how best to teach leadership, how the education should be structured, as well as a standardized definition and measure for good leadership, complicates training, assessment, and comparison of approaches in both research and education.

**Objective:**

The aim of this study is to map out the existing body of research on leadership education for physicians and medical students and identify any gaps in this literature.

**Methods:**

The scoping review will follow the Arksey and O'Malley (Arksey and O'Malley, *Int J Soc Res Methodol*. 2005; 8(1):19–32) framework, Joanna Briggs Institute methodology (JBI Manual for Evidence Synthesis—JBI Global Wiki) and PRISMA_ScR (Tricco et al., *Ann Intern Med*. 2018; 169(7):467–473). A systematic search will be conducted across the following databases: PubMed, Cochrane, Embase, Ebsco, Medline, and Google scholar. Two independent reviewers will screen titles and abstracts, then review the full texts of articles. Data will be extracted and presented in line with the review questions.

**Strengths and Limitations of this Study:**

The study will use a structured approach, as guided by Arksey and O'Malley and JBI methodology. Studies not written in English, Danish, German, Swedish, and Norwegian will be translated using available software. This review will not include a formal assessment of the study quality or meta‐analysis, as it is a scoping review.

## INTRODUCTION

Leadership is a key skill for physicians. Most doctors will take on a leading role at some point in their professional life, and their abilities in this area have been shown to affect patient outcomes.^1^ Many junior doctors will take part in activities which involve leadership,^2^ but studies show that many feel unprepared for this role.^3^

In recent years, there has been increasing interest and research into the training and development of clinical leadership, like the Medical Leadership Competency Framework in the United Kingdom,^4^ as well as attempts to use frameworks from other fields for education or evaluation of leadership.^5^, ^6^ However, there are many barriers to developing and implementing a formal leadership curriculum. These include time constraints during the education and medical students' lack of awareness of leadership as a competence to prioritize among many other skills.^5^, ^7^ Research also points to the lack of a standardized definition and measure for good leadership, which complicates training, assessment, and comparison of approaches in both research and education.^8^

In order to develop a definition and measure, as well as a formal curriculum on clinical leadership, a comprehensive assessment of the research and material on the subject is needed. This will serve as a foundation for future research, point to holes in the knowledge of leadership education, and help to establish quality education in clinical leadership.

## REVIEW QUESTIONS

1. Which types of evidence regarding clinical leadership education exist?
2. What aspects of successful clinical leadership does the literature identify?
3. How is clinical leadership taught and what are the pros and cons of these methods?
4. How is clinical leadership measured and what are the pros and cons of the methods in use?
5. How is leadership education currently structured?

## INCLUSION CRITERIA/PICO

### Participants

Medical students and physicians (all levels of education). Defined as any person who is either studying to become an allopathic^9^ medical doctor or is licensed to practice as an allopathic medical doctor. This does not include nurses or doctors licensed only to practice osteopathic^10^ medicine.

### Concept

Intervention: leadership training/education must be a primary intervention in the studies included. This includes any course, simulation training, or educational intervention with the explicit aim of improving leadership skills. These include, but are not limited to the following interventions from the EPOC Data Collection Checklist^11^:

1. Distribution of educational materials (Distribution of published or printed recommendations for clinical care, including clinical practice guidelines, audio‐visual materials, and electronic publications. The materials may have been delivered personally or through mass mailings).
2. Educational meetings (Health care providers who have participated in conferences, lectures, workshops or traineeships).
3. Educational outreach visits (Use of a trained person who met with providers in their practice settings to give information with the intent of changing the provider's practice. The information given may have included feedback on the performance of the provider(s)).

### Context

This study will focus on clinical leadership, not administrative. Any training must be aimed at the role as clinical leader. The definition of clinical refers to: ‘medical work or teaching that relates to the examination and treatment of ill people’.^12^ Clinical leadership therefore, refers to the doctors role as leader in the clinical situation, as opposed to administrative leadership: ‘of or relating to administration or an administration: relating to the management of a company, school, or other organization’.^13^

### Exclusion

Opinion pieces, viewpoints, review protocols, conceptual frameworks, conference abstracts, commentaries, conference proceedings, and book reviews will be excluded in this paper. All non‐health papers will be excluded from this review. Studies focused primarily on gender differences in leadership will be excluded.

## METHODS

### Search strategy

A search strategy will be devised following the JBI three‐step framework.

First, an initial search will be conducted based on initial keywords. Retrieved papers will be screened to find additional keywords, synonyms, and index terms. MeSH databases will be consulted to broaden the search. The search syntax will be reviewed by the librarian (AM) to ensure its quality.

Second, a full search will be conducted across six databases (PubMed, Cochrane, Embase, Ebsco, Medline, PsycInfo and Google scholar) using all identified keywords and index terms.

Third, the results of the search will be screened for duplicates.

### Selection of studies

Following the search, all identified citations will be collated and downloaded.

Titles and abstracts will be screened by two independent reviewers for assessment against the inclusion criteria. The same reviewers will read the full text of selected citations and assess it in detail against the inclusion criteria. Any disagreements that arise between the reviewers at each stage of the selection process will be resolved through discussion or with an additional reviewer. Reasons for the exclusion of full‐text sources that do not meet the inclusion criteria will be recorded and reported in the scoping review. The results of the search will be reported in full in the final scoping review and presented in a PRISMA flow diagram.

### Data extraction

Data will be extracted by two independent reviewers using a data charting tool based on the review questions. A pilot test will be conducted, where a sample of five papers will be reviewed, and the reviewers will go through the extracted data, ensuring that it is done correctly.

### Analysis and presentation

For each review question, the extracted data will be summarized qualitatively and quantitatively. Visuals, such as tables and charts, will be used to report the data synthesis if this is deemed helpful. Due to the range of literature on the topic of leadership education, the most effective way to present the data will be finally decided on once the data have been collected.

## DISCUSSION

This scoping review will aim to map out, as well as identify gaps in the existing literature on clinical leadership education. Exploring how clinical leadership education is structured and how the results of it are measured, as well as what pros and cons of these methods are, will help identify areas for improvement. It will also provide a summary of which aspects of leadership are currently taught. This can serve as groundwork for developing future courses, curricula, and training.

Limitations of this study will be the variability of methods, level of education, as well as the number of participants and measure of results, due to the lack of consistent guidelines on how clinical leadership is taught. This will most likely affect this review's efforts to make a general conclusion.

## AUTHOR CONTRIBUTIONS

N.V. conducted the initial literature search, and drafted the protocol. P.D. and A.M.M. supervised the project, provided methodological guidance, and contributed to the review and revision of the manuscript. All authors approved the final version of the manuscript.

## FUNDING INFORMATION

None.

## Data Availability

Data sharing not applicable to this article as no datasets were generated or analyzed during the current study.
